# Regulation of Mammary Stem/Progenitor Cells by PTEN/Akt/β-Catenin Signaling

**DOI:** 10.1371/journal.pbio.1000121

**Published:** 2009-06-02

**Authors:** Hasan Korkaya, Amanda Paulson, Emmanuelle Charafe-Jauffret, Christophe Ginestier, Marty Brown, Julie Dutcher, Shawn G. Clouthier, Max S. Wicha

**Affiliations:** 1 Comprehensive Cancer Center, Department of Internal Medicine, University of Michigan, Ann Arbor, Michigan, United States of America; 2 Laboratory of Molecular Oncology, Marseille Cancer Research Institute, UMR 599 Inserm/Institut Paoli – Calmettes, Marseille, France; University of Rochester Medical Center, United States of America

## Abstract

Recent evidence suggests that many malignancies, including breast cancer, are driven by a cellular subcomponent that displays stem cell-like properties. The protein phosphatase and tensin homolog (PTEN) is inactivated in a wide range of human cancers, an alteration that is associated with a poor prognosis. Because PTEN has been reported to play a role in the maintenance of embryonic and tissue-specific stem cells, we investigated the role of the PTEN/Akt pathway in the regulation of normal and malignant mammary stem/progenitor cell populations. We demonstrate that activation of this pathway, via PTEN knockdown, enriches for normal and malignant human mammary stem/progenitor cells in vitro and in vivo. Knockdown of PTEN in normal human mammary epithelial cells enriches for the stem/progenitor cell compartment, generating atypical hyperplastic lesions in humanized NOD/SCID mice. Akt-driven stem/progenitor cell enrichment is mediated by activation of the Wnt/β-catenin pathway through the phosphorylation of GSK3-β. In contrast to chemotherapy, the Akt inhibitor perifosine is able to target the tumorigenic cell population in breast tumor xenografts. These studies demonstrate an important role for the PTEN/PI3-K/Akt/β-catenin pathway in the regulation of normal and malignant stem/progenitor cell populations and suggest that agents that inhibit this pathway are able to effectively target tumorigenic breast cancer cells.

## Abstract 2

The PTEN/Akt/β-catenin pathway is important for maintaining stem or progenitor cells in normal and cancerous breast tissue and may be a promising target for effective, long-lasting cancer treatment.

## Author Summary

Healthy adult tissues are maintained through the regulated proliferation of a subset of cells known as tissue stem and progenitor cells. Many cancers, including breast cancer, also are thought to arise from and be maintained by a small population of cells that display stem cell-like properties. These so-called “cancer stem cells” may also contribute to tumor spread (metastasis), resistance to treatment, and disease relapse. Effective, long-lasting cancer treatments likely will need to target and eliminate these cancer stem cells specifically. Regulatory pathways responsible for maintaining cancer stem cells therefore may be promising targets for treatment. Breast cancers in humans frequently display abnormalities in the PTEN/PI3K/Akt pathway. We demonstrate using cell culture and a mouse model of breast cancer that stem or progenitor cells in both normal breast tissue and breast tumors are dependent for their continued growth on this pathway and on the Wnt/β-catenin pathway. We further show that the drug perifosine, which inhibits the kinase Akt, is able specifically to reduce the population of breast cancer stem or progenitor cells growing in mice. Our findings support the idea that drugs that selectively target breast cancer stem cells through the PTEN/PI3K/Akt pathway may reduce tumor growth and metastasis and result in improved patient outcomes.

## Introduction

There is increasing evidence that a variety of cancers, including those of the breast, may be driven by a component of tumor-initiating cells that retain stem cell-like properties. These properties include self-renewal, which drives carcinogenesis, as well as differentiation, which contributes to tumor cellular heterogeneity [1]. A number of signaling pathways have been found to play a role in mammary stem cell self-renewal, including Wnt, Notch, and Hedgehog [2]–[4]. In addition, the *PTEN* (phosphatase and tensin homolog deleted on chromosome 10) tumor suppressor gene, one of the most frequently mutated genes in human malignancies, has also been suggested to play a role in stem cell self-renewal [5]. PTEN acts as a lipid phosphatase to dephosphorylate phosphatidylinositol (3-5)-trisphosphate (PIP_3_), antagonizing the PI3-K/Akt pathway. Deletion of *PTEN* results in increased activation of the PI3-K/Akt pathway, which correlates with poor prognosis in breast cancer patients [6]. Furthermore, deletion or reduced expression of *PTEN* in a wide variety of human tumors is associated with resistance to conventional therapeutic agents and relapse following initial treatment [7],[8]. In prostate tumors, loss of *PTEN* expression predicts progression to invasive and metastatic disease [9]. Deletion of *PTEN* in murine models of prostate cancer results in expansion of the prostate stem/progenitor cell population and initiation of prostate tumors resembling those in humans [10]. In the hematopoietic system, *PTEN* deletion induces excessive proliferation of hematopoietic stem cells with subsequent depletion of this cell population in the bone marrow [11],[12]. This *PTEN* deficiency also results in the induction of myeloproliferative disorders that progress to leukemia [11],[12].

Recent studies have suggested that cancer stem cells, by virtue of their resistance to chemotherapy and radiation therapy, may contribute to tumor resistance and relapse [13],[14]. The PTEN/PI3-K/Akt pathway has been described as a major pathway conferring resistance to conventional therapies in multiple tumor types [15]–[17]. Using a large-scale RNA interference genetic screen, Berns et al. identified PTEN as the modulator of drug resistance in breast cancer [17]. Patients with HER2 amplified breast tumors that also contain *PTEN* deletions are resistant to Trastuzumab treatment [8]. Because cancer stem cells have been found to be resistant to radiation and chemotherapy, we postulated that the PTEN/Akt pathway may play a role in the regulation of mammary stem/progenitor cells. Thus, we examined the PI3-K/Akt pathway and characterized its downstream signaling components for their role in regulating mammary stem/progenitor cells. In the present study, we demonstrate that the PI3-K/Akt pathway plays an important role in regulating the Aldefluor-positive cell population, which is enriched in mammary stem/progenitor cells, by mediating Wnt/β-catenin signaling through phosphorylation of GSK3-β. Furthermore, we demonstrate that the Akt inhibitor perifosine is able to target normal and malignant Aldefluor-positive mammary epithelial cells in vitro and in mouse xenograft models.

## Results

### The PTEN/PI3-K/Akt/β-Catenin Pathway Is Activated in Mammospheres as Compared with Monolayer Cultures

We have previously demonstrated that primitive mammary stem/progenitor cells are enriched in vitro in floating spherical colonies termed mammospheres. Mammospheres are composed of a small number of cells with stem cell-like properties including the ability to form secondary mammospheres as well as the ability to undergo multilineage differentiation [18]. In addition, we recently reported the enrichment of mammary stem/progenitor cells within the aldehyde dehydrogenase (ALDH)-expressing cell population as assessed by the Aldefluor assay [19]. When these primitive mammary cells are cultured in the presence of serum on an adhesive substratum, they lose these primitive properties and undergo differentiation. Recent studies suggest that signal transduction pathways including the PTEN/PI3-K/Akt play a role in embryonic and tissue-specific stem cell self-renewal [10],[20],[21].

**Figure 1:**
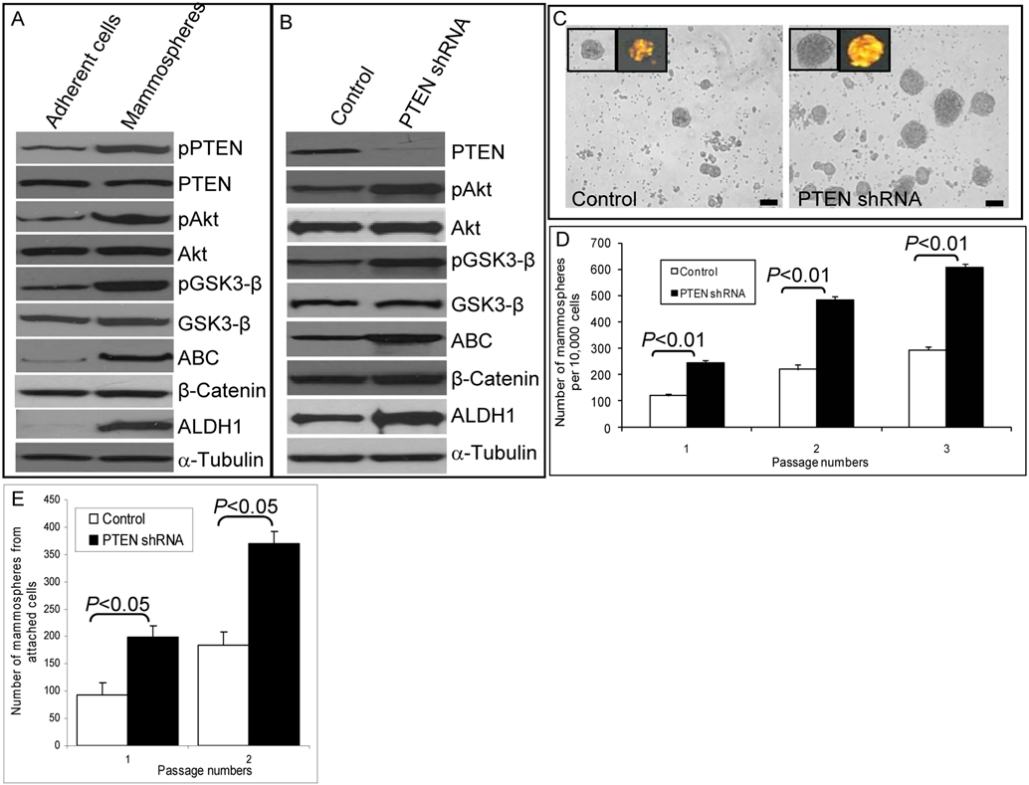
Activation of PTEN/PI3-K/Akt/GSK3-β/β-catenin signaling in mammospheres. (A) After 7–10 d of culture, mammospheres and adherent NMECs were analyzed by Western blotting for activation of the PI3-K/Akt pathway and its downstream targets. Mammospheres as compared to adherent NMEC cultures demonstrated increased phosphorylation of PTEN, Akt, GSK3-β, and activated β-catenin (ABC). Mammospheres but not the adherent cells also expressed the marker ALDH1. (B) Knockdown of PTEN expression via shRNA lentivirus infection led to further increases in phospho-Akt, phospho-GSK3-β, and activated β-catenin levels compared with DsRed lentiviral-infected control mammospheres. (C) PTEN knockdown led to an increase in the number of mammosphere forming cells. The efficiency of lentivirus infection was demonstrated by DsRed expression (inserts). (D) DsRed control and PTEN knockdown mammospheres were cultured for three passages, and the number of mammospheres generated per 10,000 cells was determined. (E) Adherent NMECs were infected with control or PTEN lentiviral constructs and maintained in attachment cultures for 7 d. The cells from these attachment cultures were assessed for their mammosphere-forming ability. As indicated, cells with PTEN knockdown generated more mammospheres than control cells Scale bars in (C) = 100 µm. Each data point in (D) and (E) represents the mean±SD of three independent experiments.

To determine whether this pathway was activated in primitive mammary cells, we compared the levels of PTEN and Akt phosphorylation and its downstream targets in normal mammary stem and progenitor cells in mammospheres compared with those in cells induced to undergo differentiation in monolayer cultures. Activation of the PTEN/PI3-K/Akt pathway was assessed by Western blotting using phospho-specific antibodies. As compared to adherent cultures, normal mammary epithelial cells (NMECs) in mammosphere cultures expressed increased Ser^380^ phosphorylation of PTEN (Figure 1A), which results in its conformational changes masking the PDZ binding domain [22]. PTEN, through its lipid phosphatase activity, antagonizes PI3-K/Akt signaling. We detected increased Akt Ser^473^ phosphorylation in mammospheres as compared with monolayer cultures, suggesting that inactivation of PTEN results in increased Akt phosphorylation in more primitive cells (Figure 1A). Akt has a number of known downstream targets including GSK3-β, which regulates the Wnt/β-catenin pathway. As compared to differentiated cells, cells in mammospheres displayed increased levels of GSK3-β phosphorylation and β-catenin activation (Figure 1A). β-catenin has been demonstrated to play an important role in the development of mammary stem cells in mouse models [23], suggesting that this pathway may also be active in human mammary stem/progenitor cells in mammospheres.

We have previously reported an enrichment of mammary stem/progenitor cells within the cell population expressing of ALDH, which can be detected by the enzymatic Aldefluor assay [19],[24]. Consistent with this, we found significantly higher ALDH expression in mammospheres as compared to attached mammary epithelial cell cultures (Figure 1A). We also analyzed activation of the Wnt/β-catenin pathway in ALDH-expressing cells. Aldefluor-positive cells showed significantly higher β-catenin activation as well as increased GSK3-β phosphorylation as compared with Aldefluor-negative cells (Figure S1), suggesting that mammary stem/progenitor cells display activation of the Wnt/β-catenin pathway.

### Activation of the PTEN/PI3-K/Akt Pathway Results in Enrichment of the Mammary Stem/Progenitor Cell Population In Vitro

To examine the functional role of the PI3-K/Akt/GSK3-β/β-catenin pathway, we used both gain-of-function and loss-of-function strategies. To activate this pathway, PTEN levels were decreased by using a PTEN shRNA DsRed-labeled lentivirus. Inclusion of the DsRed label allowed us to eliminate noninfected cells by flow cytometry. As shown in Figure 1B, we achieved greater than an 80% reduction in PTEN protein expression in mammospheres as assessed by Western blotting. Knockdown of PTEN in these cells resulted in increased levels of Akt phosphorylation, GSK3-β phosphorylation, and β-catenin activation (Figure 1B). In addition, activation of this pathway further increased expression of ALDH1 (Figure 1B).

To examine the functional role of the PTEN/PI3-K/Akt pathway in human mammary stem/progenitor cell fate, we measured the effect of PTEN knockdown on mammosphere formation. Knockdown of PTEN resulted in an increase in the number of primary and secondary mammospheres as compared to control, *p*<0.01 (Figure 1C and 1D). Furthermore, this increase was maintained upon serial passage to tertiary mammospheres (Figure 1D). To provide additional evidence that this pathway enriches for mammary stem/progenitor cells, we determined the effect of PTEN knockdown on the percentage of cells expressing ALDH as assessed by the Aldefluor assay. Primary NMECs from reduction mammoplasties contain between 4% and 9% Aldefluor-positive cells, which increases to 14%–19% in primary mammospheres, consistent with the enrichment of stem/progenitor cells when grown in suspension cultures (Figure S2A). PTEN knockdown increased the proportion of Aldefluor-positive cells (*p*<0.01) in mammospheres more than 2-fold to 37%–41% (Figure 1E). Thus, knockdown of PTEN resulted in enrichment of mammary stem/progenitor cells in vitro as determined by both mammosphere and Aldefluor assays. We previously observed that while mammary stem/progenitor cells are enriched in mammosphere cultures, they are depleted in attachment cultures (Figure S2B). Cells with PTEN knockdown maintained a higher percentage of Aldelfuor-positive cells in attachment culture (Figure S2B) as well as in suspension culture (Figure S2A). Furthermore, PTEN knockdown increased phospho-Akt expression in cells grown either in attachment or suspension cultures (Figure S2C). These results suggest that PTEN knockdown is able to enrich for the stem/progenitor cell population independent of culture conditions.

### PTEN Knockdown in NMECs Induces Morphological Changes with Features of Atypical Hyperplasia in Humanized NOD/SCID Mice

**Figure 2:**
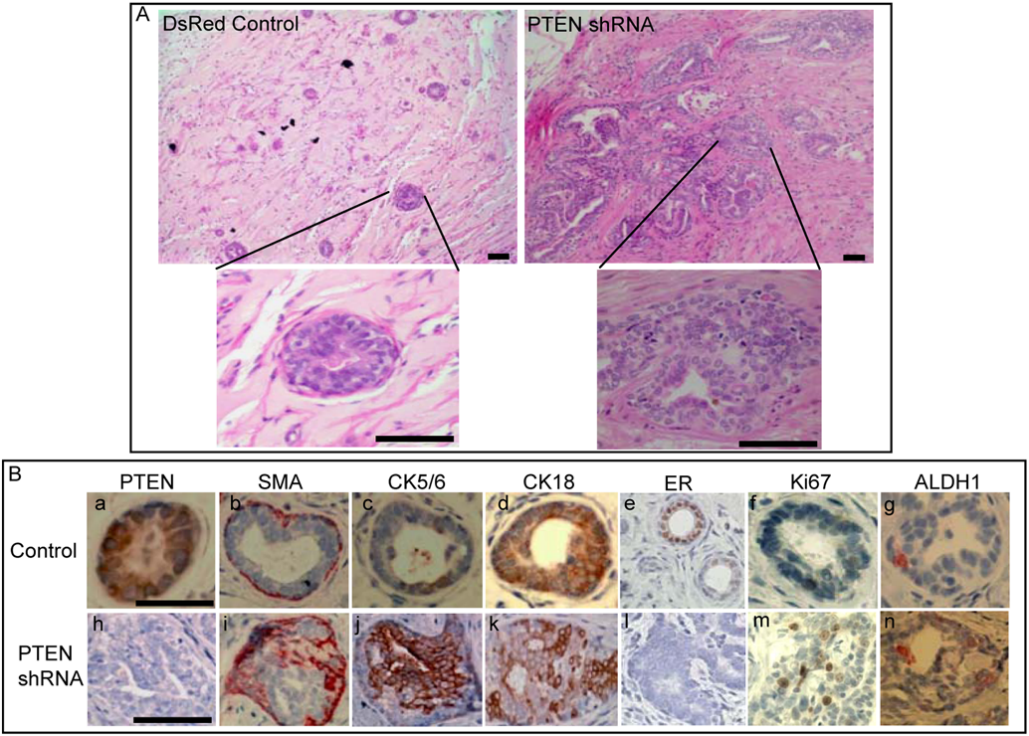
Knockdown of PTEN in NMECs generates disorganized hyperplastic lesions in humanized NOD/SCID mice. (A) Human mammary outgrowths generated from control or PTEN knockdown NMECs in humanized NOD/SCID mice exhibited an altered morphology by hematoxylin and eosin staining. (B) PTEN staining demonstrated reduced PTEN protein expression in outgrowths generated from PTEN knockdown cells as compared to the controls (a and h). Smooth muscle actin (SMA) staining revealed disorganized myoepithelial structures in PTEN knockdown outgrowths compared to an organized layer of myoepithelial cells in controls (b and i). Outgrowths with PTEN knockdown showed increased CK5/6 expression (c and j) and decreased CK18 expression (d and k), as well as a lack of ERα expression compared to control cells (e and l). PTEN knockdown outgrowths displayed increased proliferation characterized by Ki67 staining compared to controls (f and m). Increased ALDH1 expression was demonstrated in PTEN knockdown structures (g and n). Scale bars = 100 µm. Data are representative of experiments with five mice in each group.

We previously used a mouse model described by Proia and Kuperwasser [25] in which NMECs form outgrowths in NOD/SCID mice whose mammary fat pads have been humanized by the introduction of both irradiated and non-irradiated human mammary fibroblasts. We used this system to examine the effects of PTEN knockdown on mammary development. Serial dilutions of flow cytometry-sorted cells were introduced into the humanized fat pads of NOD/SCID mice. As indicated in Table S1, at all dilutions, NMECs with PTEN knockdown were more efficient in generating outgrowths than DsRed control cells. While at least 10,000 control cells were required for efficient outgrowth formation, as few as 250 PTEN knockdown cells generated outgrowths in 50% of the mice, indicating that PTEN knockdown increased the frequency of multipotent mammary stem/progenitors. In addition, we observed significant morphological alterations in structures generated by PTEN knockdown in NMECs compared to DsRed controls (Figure 2A). PTEN knockdown cells produced much larger structures that displayed significant morphologic alterations. Knockdown of PTEN was confirmed by the lack of PTEN expression in PTEN knockdown outgrowths compared to controls (Figure 2B, a and h). We used immunohistochemical staining for markers of myoepithelial, basal, and luminal epithelial cells to ascertain the effects of PTEN knockdown on cellular differentiation. Outgrowths generated by control-infected NMECs consisted of ductal structures that were characterized by a single layer of myoepithelial cells, which expressed smooth muscle actin recapitulating the architecture of normal mammary ducts in humans. In contrast, structures generated by PTEN shRNA-infected cells were characterized by gross disorganization with increased numbers of smooth muscle actin expressing myoepithelial cells distributed throughout the gland (Figure 2B, b and i). Glands produced by control cells contained only a small number of cells expressing the primitive cytokeratins 5/6, whereas the frequency of these cells was greatly increased in PTEN knockdown structures (Figure 2B, c and j). Examination of epithelial markers also revealed significant differences between structures derived from PTEN knockdown and control cells. In control structures, the majority of the luminal epithelial cells expressed the luminal marker CK18, whereas expression of this marker occurred only in a subfraction of PTEN knockdown cells (Figure 2B, d and k). Estrogen receptor (ER) was expressed in luminal epithelial cells in structures generated from DsRed control cells, but not in structures derived from PTEN knockdown cells (Figure 2B, e and l). Furthermore, structures with PTEN knockdown displayed significant increases in proliferating cells as determined by Ki67 expression (Figure 2B, f and m). Consistent with the in vitro experiments, PTEN knockdown also increased the proportion of cells expressing ALDH1 (Figure 2B, g and n). These experiments confirm and extend the in vitro findings and suggest that in addition to resulting in enrichment of the stem/progenitor cell pool, activation of the PTEN/PI3-K/Akt pathway affects cellular growth and differentiation. This results in the generation of cells displaying increased proliferation, with aberrant differentiation resulting in increased expression of primitive and basal markers and decreased expression of luminal epithelial markers. All of these histopathologic features are characteristic of atypical ductal hyperplasia, a premalignant lesion that may progress to invasive breast cancer.

### Inhibition of PI3-K/Akt Signaling Suppresses Mammosphere Formation In Vitro and Generation of Outgrowths in NOD/SCID Mouse

**Figure 3:**
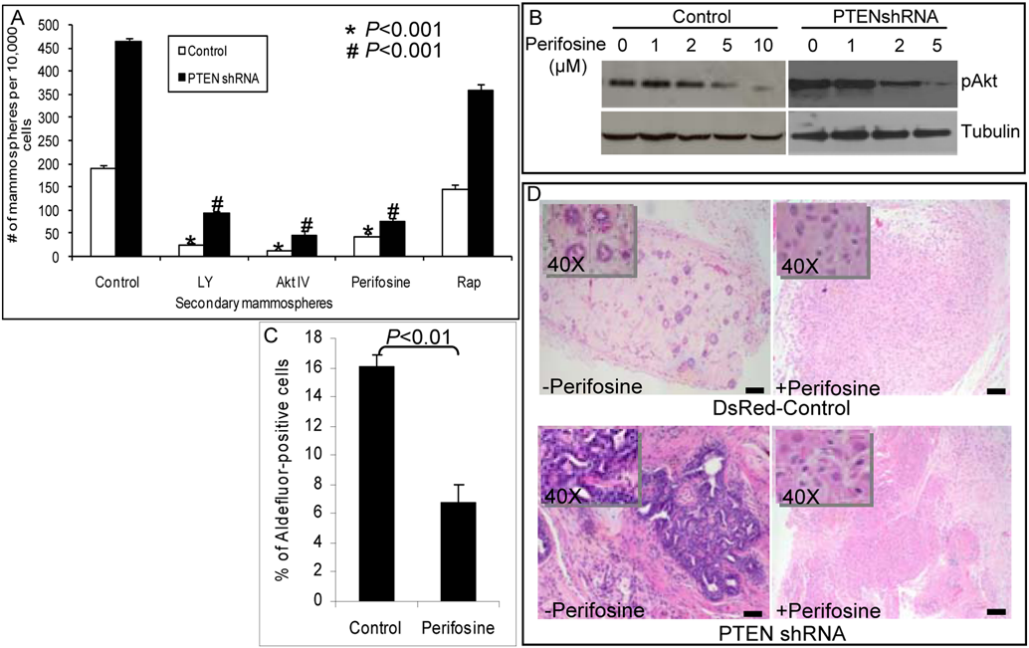
Effect of PI3-K/Akt inhibitors on mammosphere formation, ALDH1 expression, and mammary development in NOD/SCID mice. (A) Treatment of mammospheres with the PI3-K inhibitor LY294002 (1 µM), Akt inhibitor IV (2 µM), or perifosine (5 µM) inhibited mammosphere formation in control cells, whereas PTEN knockdown NMECs were sensitive to lower doses of these inhibitors: LY294002 (0.5 µM), Akt inhibitor IV (1 µM), or perifosine (2 µM). In contrast, the mTOR inhibitor rapamycin (0.5 µM) had little effect on these cells. (B) Perifosine dose response was tested in both control and PTEN knockdown cells. As indicated, PTEN knockdown cells with higher Akt activity are more sensitive to perifosine than control cells with an IC_50_ of 5 µM. (C) The effect of perifosine on the Aldefluor-positive population of NMECs was measured by the Aldefluor assay. Treatment of NMECs with perifosine over 5 d reduced the Aldefluor-positive population in primary mammospheres by more than 50%. (D) Perifosine treatment of mice implanted with control or PTEN knockdown NMECs completely blocked the formation of outgrowths in NOD/SCID humanized fat pads compared to saline-treated control mice. High magnification (insets) shows persistence of the inoculated cells. Scale bars = 100 µm. Data represent the mean±SD of three independent experiments.

To further characterize the pathways regulating mammary stem/progenitor cell self-renewal, we used inhibitors of PI3-K/Akt signaling as well as its downstream targets, GSK3-β and mammalian target of rapamycin (mTOR). As demonstrated in Figure 3A, treatment of NMECs with the PI3-K inhibitor LY294002 or the Akt inhibitor IV or perifosine reduced the number of both primary (unpublished data) and secondary mammospheres (*p*<0.001). Furthermore, primary mammospheres treated with inhibitors of PI3-K or Akt completely failed to form tertiary mammospheres (unpublished data). To determine whether the mTOR pathway, which is downstream of Akt signaling, plays a role in this process, we examined the effect of rapamycin, an inhibitor of this pathway, on mammosphere formation. As shown in Figure 3A and Figure S2, rapamycin had little effect on secondary mammosphere formation, suggesting that mTOR was not responsible for mediating the effects of Akt signaling. In contrast to rapamycin, PI3-K and Akt inhibitors suppressed the formation of secondary mammospheres in both control and PTEN knockdown NMECs, supporting the importance of this pathway in mammary stem/progenitor cell self-renewal (Figure 3A). To determine whether PTEN knockdown affected cellular sensitivity to Akt inhibition, we performed dose response studies with perifosine. As shown in Figure 3B, 2 µM perifosine inhibited Akt phosphorylation by more than 50% in PTEN knockdown cells while having no demonstrable effect on control cells. Consistent with the effects on mammosphere formation, the Akt inhibitor perifosine significantly reduced the proportion of Aldefluor-positive cells in mammospheres (Figure 3C). The effect of perifosine on PI3-K/Akt signaling has previously been reported [26],[27]. In that study, the authors found that 10 µM perifosine did not have any inhibitory effect on the MAPK pathway [27]. We also failed to detect an effect of perifosine on MAPK activity in our system (unpublished data).

To determine whether the PI3-K/Akt pathway was also critical for mammary development in vivo, we examined the effect of the Akt inhibitor perifosine on the development of human mammary structures generated by control DsRed infected or PTEN knockdown NMECs. NOD/SCID mice implanted with control or PTEN knockdown NMECs were treated with intraperitoneal injections of perifosine (30 mg/kg) 4 days a week for five weeks or a saline control. Consistent with previous experiments, control DsRed NMECs generated mammary ductal structures with normal morphology, PTEN knockdown NMECs generated ductal hyperplasias in saline-treated control mice. In contrast, administration of perifosine completely inhibited outgrowth formation by both control and PTEN knockdown NMECs (Figure 3D). These results further support the in vitro experiments by demonstrating a critical role for Akt signaling in normal mammary development.

### Effects of PI3-K/Akt on Mammary Stem/Progenitor Cells Are Mediated by GSK3-β/β-Catenin Signaling

**Figure 4:**
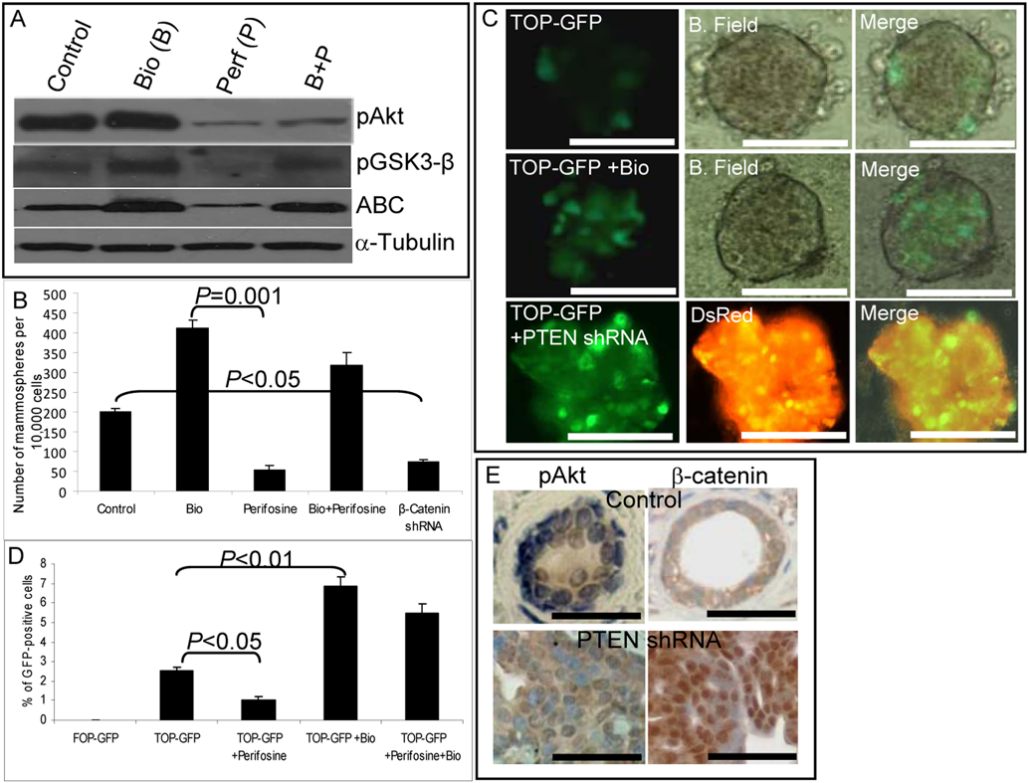
PTEN regulates β-catenin activity in mammary stem/progenitor cells. (A) Effects of the GSK3-β inhibitor (Bio) and the Akt inhibitor (perifosine) on activation of Akt/GSK3-β/β-catenin signaling as assessed by Western blotting using phospho-specific antibodies. Perifosine inhibits pAkt, pGSK3-β, and activated β-catenin expression. The GSK3-β inhibitor Bio restores β-catenin activation even in the presence of perifosine. (B) After 5 d treatment of primary mammospheres with either 10 µM perifosine or 0.5 µM Bio alone or in combination, cells were dissociated and passaged to form secondary mammospheres. Bio treatment increased the number of secondary mammospheres more than 2-fold, whereas perifosine treatment or down-regulation of β-catenin via infection with an shRNA lentivirus decreased the number of secondary mammospheres by more than 50%. Bio reversed the inhibitory effect of perifosine. (C) To monitor β-catenin activity, TOP-GFP reporter lentivirus-infected mammospheres were treated with Bio or co-transfected with PTEN shRNA. Control mammospheres cultured for 7 d contained one–four GFP-positive cells. The proportion of GFP-positive cells was increased more than 2-fold by Bio treatment. Knockdown of PTEN also resulted in more than a 2-fold increase in the proportion of GFP-positive cells. (D) TOP-GFP infected mammospheres were treated for 5 d with indicated compounds either alone or in combination and analyzed by flow cytometry. Perifosine treatment decreased the proportion of GFP-positive cells by more than 50%, whereas Bio treatment increased them more than 2-fold. The inhibition produced by perifosine was abrogated when the mammospheres were also treated with Bio. (E) Outgrowths generated in NOD/SCID mice from PTEN shRNA lentivirus-infected cells displayed increased phospho-Akt expression as well as increased nuclear β-catenin localization as compared to control outgrowths. Scale bars = 100 µm. Data represent the mean±SD of three independent experiments.

Activated Akt has been demonstrated to be capable of phosphorylating and inactivating GSK3-β, leading to nuclear translocation and activation of β-catenin [28],[29]. In addition, Akt can directly phosphorylate β-catenin, an event which further facilitates its nuclear translocation [21]. Since Wnt signaling through β-catenin has been shown to play a role in mammary development and stem cell self-renewal, we examined whether Akt effects on mammary stem/progenitor cells were mediated by this pathway. To examine the role of Wnt/β-catenin signaling, we used the GSK3-β inhibitor 6-bromoindirubin-3′-oxime (Bio), which has been shown to be able to maintain pluripotency of human and mouse embryonic stem cells through activation of β-catenin signaling [30]. We first confirmed that addition of Bio to NMECs resulted in phosphorylation of GSK3-β with subsequent activation of β-catenin (Figure 4A). To determine the biological relevance of this pathway, we examined the effects of Bio on mammosphere formation. As shown in Figure 4B, the addition of Bio increased mammosphere formation 2-fold. In addition, the reduction of mammosphere formation induced by perifosine was reversed by the addition of Bio (Figure 4B). Reversal of inhibition occurred despite the persistent inhibition of Akt activity in the presence of both compounds (Figure 4A). To confirm the importance of β-catenin signaling in stem/progenitor cell self-renewal, we knocked down β-catenin expression by using an shRNA lentivirus (Figure S3). Lentiviral shRNA-mediated down-regulation of β-catenin expression in NMECs reduced the number of mammospheres by approximately 70% (Figure 4B). These experiments suggest that the effects of Akt on mammary stem/progenitor cell self-renewal are mediated through GSK3-β/β-catenin signaling.

To provide further evidence for cross-talk between Akt signaling and the Wnt pathway, we used a LEF-1/TCF reporter system to monitor β-catenin transcriptional activity in PTEN knockdown or Bio-treated NMECs. NMECs were infected with a LEF-1/TCF reporter driving GFP (TOP-GFP) or a control reporter with mutated LEF-1/TCF binding sites (FOP-GFP). Approximately 2–5% of cells in mammospheres expressed GFP (Figure 4C), whereas FOP-GFP-infected NMECs did not express GFP (Figure 4D). Bio treatment increased the percentage of GFP-positive cells in mammospheres more than 2-fold, confirming that GSK3-β plays a role in nuclear translocation and activation of β-catenin (Figure 4C and 4D). In addition, when cells were co-infected with lentiviral PTEN shRNA and TOP-GFP, the percentage of GFP-expressing cells increased more than 2-fold (Figure 4C). To confirm that Akt regulates mammary stem/progenitor cells by activating β-catenin signaling, TOP-GFP infected cells were treated with perifosine or Bio alone or in combination. Perifosine treatment reduced the proportion of GFP-positive cells by more than 50%, whereas Bio treatment increased the number of GFP-expressing cells more than 2-fold as assessed by flow cytometry (Figure 4D). Bio reversed the effect of perifosine when mammospheres were treated with a combination of perifosine and Bio (Figure 4D). The role of β-catenin signaling in mammary stem/progenitor cells was further investigated by examining the β-catenin activity and nuclear localization in Aldefluor-positive and -negative populations. As shown in Figure S1, Aldefluor-positive but not Aldefluor-negative cells displayed GSK3-β phosphorylation and nuclear β-catenin localization.

To extend these observations to the mouse models, we examined the expression and localization of phospho-Akt and β-catenin in mammary outgrowths generated from control and PTEN knockdown NMECs. As shown in Figure 4E, control outgrowths contained cells with low levels of phospho-Akt and membranous β-catenin staining. In contrast, outgrowths generated from PTEN shRNA lentivirus-infected cells demonstrated increased phospho-Akt expression and nuclear β-catenin localization (Figure 4E). Together, these experiments demonstrate that Akt effects on mammary stem/progenitor cells are mediated by GSK3-β/β-catenin signaling.

### Akt Activation Enriches the Tumorigenic Stem/Progenitor Cell Population in Breast Cancer Cell Lines and Tumor Xenografts

**Figure 5:**
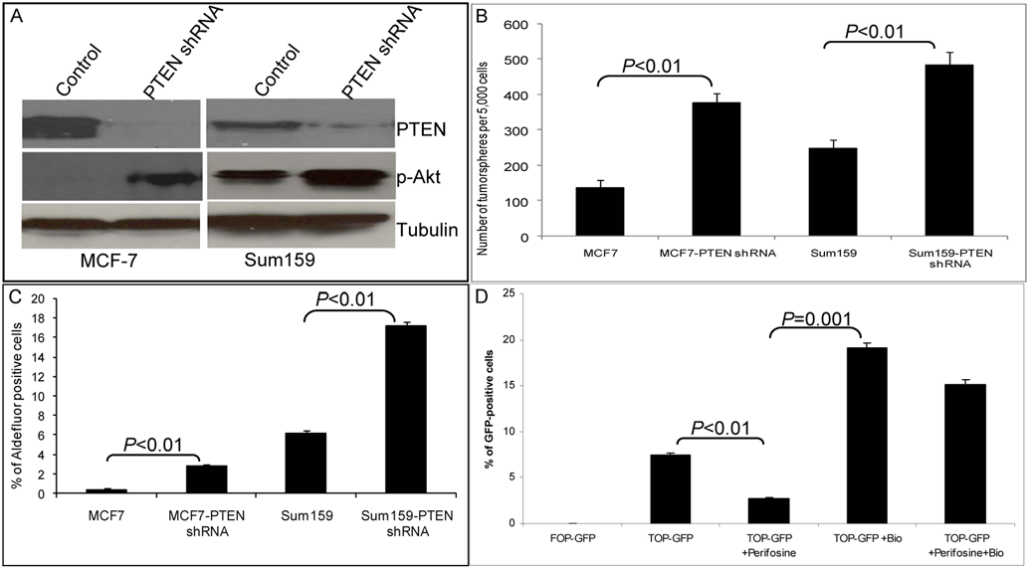
Knockdown of PTEN in breast cancer cell lines results in enrichment of breast cancer stem/progenitor cells via the Akt/GSK3-β/β-catenin pathway. (A) PTEN knockdown in MCF7 or SUM159 cells resulted in increased Akt phosphorylation as assessed by Western blotting. (B) PTEN knockdown resulted in increased secondary tumorsphere formation in MCF7 and SUM159 cells. (C) PTEN knockdown secondary tumorspheres contained an increased proportion of Aldefluor-positive cells as compared with the tumorspheres from parental lines. (D) Flow cytometry analyses of TOP-GFP-infected SUM159 tumorspheres treated with indicated inhibitors. Perifosine treatment decreased the proportion of GFP-positive cells by more than 50%, whereas Bio treatment increased the proportion of GFP-positive cells more than 2-fold and reversed the inhibitory effect of perifosine. Data represent the mean±SD of three independent experiments.

By using primary mammary carcinoma xenografts, we previously demonstrated that cancer cells with stem cell-like properties were contained within the Aldefluor-positive population [19]. Recent studies have suggested that established breast cancer cell lines also contain subpopulations with stem cell-like properties. In MCF7 and SUM159 cell lines, we previously demonstrated that only the Aldefluor-positive populations were able to form tumors capable of serial passaging in NOD/SCID mice [31]. To determine whether the PTEN/PI3-K/Akt pathway played a similar role in the regulation of malignant mammary stem/progenitor cells to that of normal mammary stem/progenitor cells, we determined the effect of knocking down PTEN expression on the cancer stem/progenitor cell populations in these cell lines. As shown by Western blotting (Figure 5A), control MCF7 cells expressed PTEN, but not phospho-Akt. Knockdown of PTEN with an shRNA lentivirus resulted in Akt activation (Figure 5A). SUM159 cells display a baseline level of phospho-Akt expression, which was further enhanced by PTEN knockdown (Figure 5A). To determine whether reduction of PTEN expression affected the mammary cancer stem/progenitor cell components of these cell lines, we used tumorsphere and Aldefluor assays. As shown in Figure 5B, knockdown of PTEN increased tumorsphere formation in both MCF7 and SUM159 breast carcinoma cells. Furthermore, this knockdown resulted in more than a 2-fold increase in the Aldefluor-positive population (*p*<0.01) in these cell lines (Figure 5C). We confirmed that PTEN knockdown in MCF7 xenographs resulted in increased Akt phosphorylation (Figure S4B). Subsequently we determined whether the increase in the cancer stem/progenitor cell population resulting from PTEN knockdown resulted in increased tumorigenicity. As shown in Figure S4A and S4C, PTEN knockdown in MCF7 or SUM159 cells increased their tumorigenicity in NOD/SCID mice. Since Zhou et al. previously reported that the side population (SP) in MCF7 contained tumor-initiating cells, we examined the overlap between the SP and Aldefluor-positive populations. As demonstrated in Figure S4, we found a 2-fold enrichment of Aldefluor-positive population in the MCF7 SP population as compared to non-SP cells. This suggests that the Aldefluor and SP assays detect distinct, but partially overlapping, cell populations. We previously reported that tumorigenicity in these cell lines is mediated by the Aldefluor-positive population, which is enriched for tumorigenic cancer stem/progenitor cells [31]. Thus, these results suggest that Akt activation increases tumorigenicity through effects on the cancer stem/progenitor cell population. We next used the TOP-GFP reporter system to determine whether Akt signals through the Wnt/β-catenin pathway in breast carcinoma cells. Perifosine treatment of TOP-GFP-infected SUM159 breast cancer cells resulted in a significant decrease in GFP-positive cells (*p*<0.01), whereas treatment of these cells with Bio increased the proportion of GFP-positive cells 3-fold, *p*<0.01 (Figure 5D). As was the case for normal mammary cells, Bio treatment was able to reverse the effect of perifosine in these mammary carcinoma cells (Figure 5D).

### Perifosine Treatment Reduces the Proportion of Aldefluor-Positive Cells in Breast Carcinoma Xenografts

**Figure 6:**
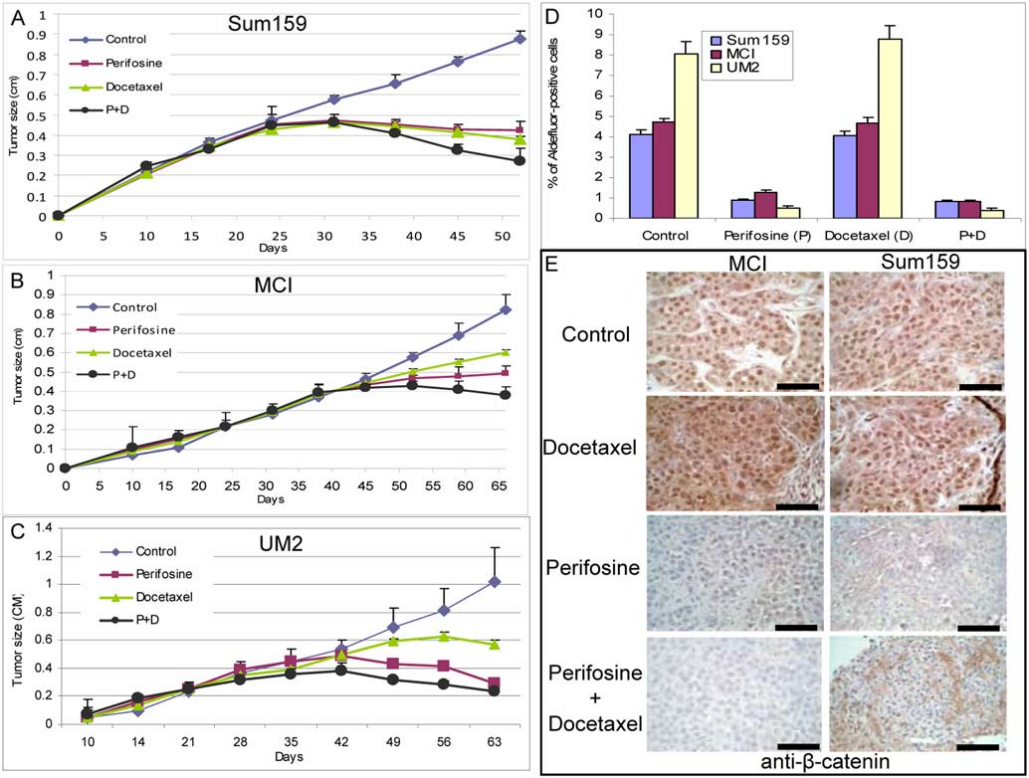
Perifosine targets the Aldefluor-positive cell population in breast cancer xenografts. (A–C). 50,000 cells from SUM159 cell line or two breast cancer xenografts, MCI, or UM2 were injected into NOD/SCID mice and the tumor size was monitored. When the tumors were approximately 4 mm, perifosine (30 mg/kg) or docetaxel (10 mg/kg), or a combination of the two agents were administered intraperitoneally once per week. Tumor size before and during the course of each indicated treatment is shown. (D) ALDH activity was assessed by the Aldefluor assay. Control or docetaxel-treated SUM159, MCI, or UM2 tumor xenografts contained a fraction of cells ranging from 4–8%, which displayed Aldefluor activity. In contrast, perifosine treatment alone or in combination with docetaxel produced a 75–90% decrease in the proportion of cancer stem/progenitor cells as assessed by the Aldefluor assay. (E) Effect of treatment on β-catenin expression. Control and docetaxel-treated tumors displayed abundant nuclear β-catenin expression. This was significantly reduced by perifosine treatment. Scale bars = 100 µm. Data represent the mean±SD of experiments with five mice in each group.

To determine whether Akt also drives the tumorigenic Aldefluor-positive cell population in vivo, we examined the effects of the Akt inhibitor perifosine on the growth of SUM159 breast cancer cells in NOD/SCID mouse xenografts. SUM159 cells are more tumorigenic than MCF7 cells, which may relate to their higher level of Akt phosphorylation. In addition, to extend this study to primary tumor xenografts, we examined the effects of this inhibitor on two different breast cancer xenografts, MC1 and UM2, in which we previously demonstrated that the tumorigenic cells are contained within the Aldefluor-positive population [19]. We compared the effects of perifosine alone or with docetaxel, a chemotherapeutic agent that is commonly used to treat human breast cancers. As shown in Figure 6, both perifosine and docetaxel significantly inhibited tumor growth in all three xenografts compared with saline-treated controls. Furthermore, the combination of perifosine and docetaxel reduced tumor size compared with either treatment alone (Figure 6A–6C). After 4 wk of treatment, animals were killed, and cells in the residual tumors were analyzed by the Aldefluor assay. Treatment of mice with docetaxel had no significant effect on the percentage of Aldefluor-positive cells. In contrast, perifosine either with or without docetaxel significantly (*p* = 0.001) reduced the Aldefluor-positive population by over 75% in Sum159 and MCI (Figure 6A and 6B) and over 90% in UM2 xenografts (Figure 6C). (Flow cytometry data are presented in Figure S6A–S6C).

To determine whether these treatments affected the Wnt/β-catenin pathway, we examined their effects on β-catenin expression and cellular localization. Nuclear β-catenin was detected in the majority of cells from control and docetaxel-treated animals. In contrast, tumors treated with perifosine or the combination of perifosine and docetaxel showed a significant reduction in β-catenin expression that was largely localized in the cytoplasm or plasma membrane rather than nucleus (Figure 6D).

**Figure 7:**
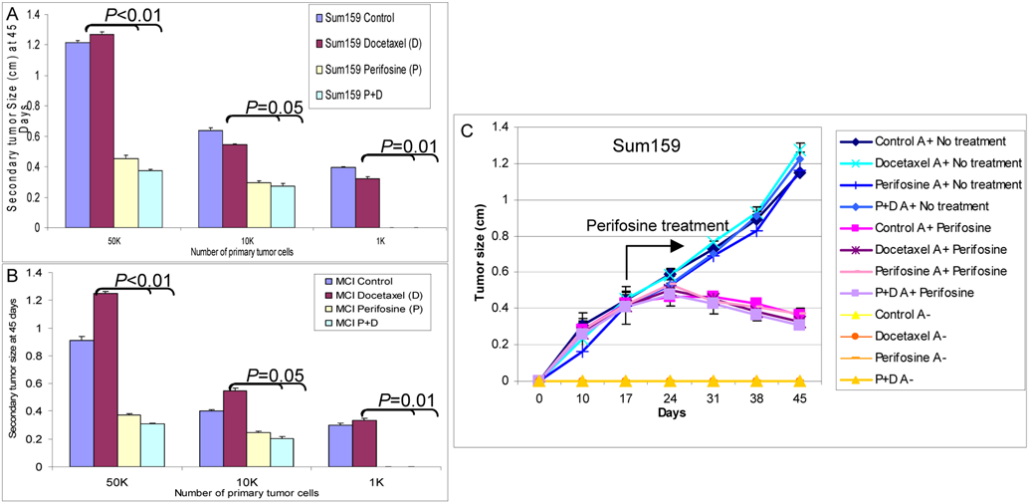
Perifosine treatment reduces the tumorigenic cell population as assessed by reimplantation in secondary NOD/SCID mice. (A and B). Serial dilutions of cells obtained from primary Sum159 (A) or MCI (B) tumors treated with saline control, perifosine, docetaxel, or both were implanted into secondary NOD/SCID mice. Control and docetaxel-treated primary tumors formed secondary tumors at all dilutions. In contrast, primary tumors treated with perifosine alone or in combination with docetaxel demonstrated growth delay with secondary tumors that were three times smaller in size than the control or docetaxel-treated tumors. Furthermore, 1,000 cells from perifosine or perifosine- plus docetaxel-treated primary tumors failed to form any secondary tumors, whereas tumors grew in mice inoculated with an equivalent number of cells from control or docetaxel-treated tumors. (C) Primary treated tumors were sorted by the Aldefluor assay and 5×10^3^ cells from each of the Aldelfuor-positive and Aldelfuor-negative populations were reimplanted in secondary NOD/SCID mice. Only Aldefluor-positive cells generated secondary tumors that remained sensitive to perifosine treatment. Data represent the mean±SD of experiments with five mice in each group.

Although cancer stem cells are enriched within the Aldefluor-positive cell population, a more definitive assay for breast cancer stem cells involves their ability to self-renew as determined by tumorigenicity in NOD/SCID mice. We therefore determined the ability of serial dilutions of cells obtained from control or treated tumors to form secondary tumors in NOD/SCID mice. Figure 7A and 7B demonstrate that tumor cells derived from docetaxel-treated or control animals showed similar tumor regrowth at all dilutions in secondary NOD/SCID mice. In contrast, perifosine treatment with or without chemotherapy significantly reduced tumor growth in secondary recipients (Figure 7A and 7B). When equal numbers of cells were injected, those from perifosine treated animals showed a 2–3-fold reduction (For 5×10^4^ cells; *p*<0.01, for 1×10^4^ cells; *p* = 0.05, and for 1×10^3^ cells; *p* = 0.01) in tumor growth compared to cells from chemotherapy treatment alone or control animals (Figure 7A and 7B and Figure S6). Furthermore, in contrast to cells obtained from docetaxel treatment or control animals, 1,000 cells from perifosine or the combination of perifosine- and docetaxel-treated animals failed to produce tumors in secondary mouse xenografts (Figure 7A and 7B). The kinetics of secondary tumor growth at different inoculation dilutions are shown in Figure S7A and S7B. Thus, the Aldefluor assay as well as tumor regrowth experiments indicate that perifosine is able to target and reduce the Aldefluor-positive population, whereas one of the most commonly used chemotherapeutic agents, docetaxel, failed to do so.

To determine whether perifosine treatment selected for a resistant population of tumor stem/progenitor cells, cells from tumors remaining after perifosine or docetaxel treatment were sorted by the Aldefluor assay and reimplanted in secondary NOD/SCID mice. As we have previously reported [19], only Aldefluor-positive cells were tumorigenic in NOD/SCID mice (Figure 7C). Furthermore secondary tumors generated from perifosine- or docetaxel-treated tumors were as sensitive to perifosine as were primary tumors (Figure 7C). Together these studies indicate that perifosine is able to significantly reduce the tumorigenic cell population without selecting for resistant cells.

## Discussion

The cancer stem cell hypothesis holds that cancer arises in tissue stem or progenitor cells through dysregulation of the self-renewal process [32],[33]. This process generates tumors organized in a cellular hierarchy that are driven by “cancer stem cells,” which are capable of self-renewal as well as differentiation, generating the bulk of the tumor. Although considerable progress has been made in identifying “cancer stem cells” in a variety of hematologic and solid human malignancies, the pathways that drive transformation of these cells are poorly understood. We and others have suggested that carcinogenesis may involve dysregulation of the normally tightly regulated process of stem cell self-renewal coupled to aberrant differentiation of progeny cells [1].

The *PTEN* tumor suppressor gene is one of the most frequently dysregulated genes in breast cancer. Mouse genetic studies reveal that *PTEN* is essential for embryonic development [5], with heterozygous mice developing tumors in several organs including the breast [5],[34]. Germline mutations of *PTEN* cause cancer predisposition and a rare developmental disease called Cowden syndrome, which is associated with an increased incidence of breast cancer [35]. Humans with germline *BRCA1* mutations may also develop microdeletions in the *PTEN* gene [36]. There is also accumulating evidence that *PTEN* may play a role in stem cell self-renewal [37].

We have used both in vitro systems and mouse models to demonstrate an important role for the PTEN/PI3-K/Akt/β-catenin pathway in regulating both normal and malignant mammary stem/progenitor cells. Compared with differentiated normal mammary epithelial cells in monolayer cultures, mammospheres displayed significantly higher levels of Akt phosphorylation. This was accompanied by increased GSK3-β phosphorylation and β-catenin activation, suggesting that the β-catenin signaling pathway may have a role in maintaining mammary stem/progenitor cell population. The Wnt/β-catenin pathway has previously been shown to play a role in mammary stem cell function in transgenic mouse models [38]. To examine the role of these signaling pathways in mammary stem cell function, we used both gain-of-function and loss-of-function approaches. Knockdown of PTEN using a PTEN shRNA lentiviruses resulted in an enrichment of mammary stem/progenitor cells in vitro as evidenced by mammosphere formation and by the expression of ALDH as assessed by the Aldefluor assay [19]. Increases in percentage of Aldelfuor-positive cells and mammosphere formation were seen in PTEN knockdown cells even when they were initially cultured in attached conditions, indicating that PTEN knockdown maintains a higher proportion of mammary stem/progenitors. We have previously demonstrated an enrichment of stem/progenitor cells within the Aldefluor-positive cell population as well as in mammospheres [19],[39]. We next examined the effects of Akt activation via PTEN knockdown on the formation of mammary outgrowths in NOD/SCID mice whose mammary glands were humanized by the introduction of human mammary fibroblasts [19],[25]. Control structures generated from DsRed-infected NMECs were composed of CK18+ epithelial cells, a portion of which also expressed ERα, surrounded by a single layer of smooth muscle actin expressing myoepithelial cells. These structures closely resembled those found in normal human mammary ducts. In contrast, PTEN shRNA lentivirus-infected cells generated hyperplastic structures exhibiting gross tissue disorganization. This was characterized by an increase in cells expressing primitive cytokeratins 5 and 6 and a decrease in cells expressing the luminal marker CK18, indicating an expansion of primitive cells. This was further evidenced by a lack of ERα expression. Furthermore, these disorganized structures contained an increased proportion of proliferating cells, as evidenced by Ki67 expression and an expansion of ALDH1-expressing cells. All of these histologic characteristics resembled those of atypical ductal hyperplasia, one of the most common premalignant lesions in humans that is believed to be a precursor of DCIS (ductal carcinoma in situ) and invasive ductal carcinoma [40]. Interestingly, inactivating mutations of *PTEN* have been reported to be present in these preneoplastic lesions in humans [41]. Our studies provide a potential molecular explanation for these findings by suggesting that PTEN knockdown and subsequent Akt activation may regulate mammary stem cell self-renewal with an accompanying alteration in cellular differentiation, events which may be important during the initiating stages of carcinogenesis.

Utilizing the SP from MCF7 cells, Zhou et al., recently suggested that the PTEN/mTOR/STAT3 pathway is required for the maintenance of breast cancer stem cells [42]. We detected a 2-fold enrichment of Aldefluor-positive cells in the SP population, suggesting that these assays detect distinct, although partially overlapping, cell populations. We found no evidence for mTOR regulation of normal or malignant mammary stem/progenitor cells. There might be several explanations for this discrepancy. A number of reports have indicated that inhibition of mTOR results in the activation of Akt through a positive-feedback loop [43]–[46]. Furthermore, recent studies have shown that mammary stem cells are not contained within the SP population [47]. In contrast to the report by Zhou et al., we used both pharmacologic and genetic approaches to elucidate the downstream signaling pathways of Akt in both normal and malignant mammary stem/progenitor cells. Treatment of NMECs with the PI3-K inhibitor Ly294002, or the Akt inhibitors IV and perifosine, reduced mammosphere formation and the Aldefluor-positive cell population. The effect of perifosine on the PI3-K/Akt pathway has previously been described [26],[48]. It has also been reported that Akt may regulate Wnt signaling through Akt phosphorylation and inactivation of GSK3-β, which in turn mediates β-catenin degradation [28] or, directly by phosphorylating β-catenin on serine 552, promoting the nuclear translocation of β-catenin [21]. Thus, by these two mechanisms, Akt activation promotes the activation and accumulation of nuclear β-catenin [49]. We demonstrated in mammospheres that Akt phosphorylation was associated with increased phosphorylation of GSK3-β and activation of β-catenin. GSK3-β targets β-catenin for ubiquitin-mediated degradation through phosphorylation of its N-terminal serine and threonine residues [50]. To demonstrate the importance of β-catenin in mammosphere formation, we used a β-catenin shRNA lentivirus to knock down β-catenin expression. This resulted in significantly reduced mammosphere formation. Furthermore, we demonstrated that the GSK3-β inhibitor Bio rescued the effects of Akt inhibition on mammosphere formation. To examine more directly the activation of the Wnt pathway, we used a TOP-GFP reporter system that is activated by β-catenin signaling. The Akt inhibitor perifosine significantly reduced the proportion of TOP-GFP expressing cells, an effect that was reversed by Bio. The relevance of these findings to mammary development in vivo was determined by examining the expression and cellular localization of phospho-Akt and β-catenin in mammary outgrowths generated from control and PTEN knockdown NMECs. While cells from control outgrowths showed minimal phospho-Akt expression and membranous β-catenin localization, outgrowths from PTEN shRNA lentivirus-infected cells demonstrated increased phospho-Akt expression and nuclear β-catenin localization. The importance of this pathway was demonstrated by the ability of the Akt inhibitor perifosine to completely block mammary development in the mouse model. Together these in vitro and mouse experiments suggest that the effects of Akt on mammary stem/progenitor cells are mediated by GSK3-β phosphorylation and β-catenin activation.

To determine whether the PTEN/Akt/β-catenin signaling pathway also plays a role in the regulation of malignant mammary stem/progenitor cells, we used both breast cancer cell lines and a primary tumor xenograft. Although Hollestelle et al. reported an activating *PIK3CA* mutation in MCF7 cells, they did not determine whether this mutation resulted in activation of the PI3K/Akt pathway [51]. In contrast, Neve et al. screened 38 breast cancer cell lines including MCF7 for various pathways and found that MCF7 cells displayed significantly low levels of the PI3/Akt activation when compared with other cell lines [52]. This was confirmed in a recent report demonstrating that despite harboring a *PIK3CA* helical mutation, MCF7 cells displayed a low level of AKT phosphorylation [53]. In addition, these authors found no correlation between similar activating mutations of *PIK3CA* in primary tumors and patient survival or the chemoresistance [53]. Most importantly, they found no evidence that these mutations in *PIK3CA* resulted in activation of Akt pathway as compared with the wild-type *PIK3CA,* but there was a strong correlation between *PTEN* inactivating mutation and activation of Akt signaling [53]. In agreement with latter reports, we found that PTEN knockdown in MCF7 or SUM159 resulted in activation of PI3-K/Akt pathway. We demonstrated that activation of this pathway through knockdown of PTEN significantly increased tumorsphere formation and the ALDH-expressing cell population, indicating an enrichment of cancer stem/progenitor cells. Moreover, enrichment of the cancer stem/progenitor cell population directly correlated with increased tumorigenicity. Since this population mediates tumorigenesis [31], it suggests that Akt activation enhances tumorigenesis through effects on the cancer stem/progenitor cell population. Furthermore, as was the case for NMECs, we demonstrated that Akt effects on mammary carcinoma stem/progenitor cells are mediated by Wnt/β-catenin signaling. By using the TOP-GFP reporter system, we found that inhibition of Akt signaling significantly reduced the number of GFP-positive cells, an effect that was rescued with the GSK3-β inhibitor Bio. These findings are in agreement with the findings of Li et al., who reported that Wnt-induced mouse mammary tumors show expansion of stem/progenitor population as characterized by increased stem cell markers [54]. Furthermore the Wnt/β-catenin pathway has been shown to play a role in mediating the radiation resistance of mouse mammary progenitor cells [55].

In addition to its effects on the stem/progenitor cell population, Akt has been demonstrated to play a role in chemoresistance [15]–[17]. Recent evidence utilizing in vitro systems [56], animal models [13], and clinical trials have suggested that breast cancer stem cells are relatively resistant to both radiation and chemotherapy [57]. Improved clinical outcomes may require the development of strategies that are able to target this cancer stem cell population. The demonstration that Akt signaling plays a prominent role in stem cell self-renewal makes it an attractive target for such strategies. We used the Akt inhibitor perifosine, an orally bioactive alkylphospholipid, to determine its effects on Aldefluor-positive cell population. We demonstrate both in in vitro and in mouse xenograft models that perifosine is able to target the Aldefluor-positive tumorigenic cell population as determined by the Aldefluor assay and by reduced tumorigenicity upon serial transplantation. Furthermore, tumorigenic Aldefluor-positive cells remaining after perifosine treatment generated secondary tumors that were still sensitive to perifosine. In contrast, the chemotherapeutic agent docetaxel, although capable of causing tumor regression, failed to target the Aldefluor-positive cell population. If cancer stem cells indeed contribute to tumor resistance and relapse, then the addition of agents that are capable of targeting these cells may increase the clinical efficacy of current therapies. Our studies identify inhibitors of the PI3-K/Akt/Wnt signaling pathway as potential agents for therapeutic targeting of cancer stem cells.

## Materials and Methods

### Cell Lines and Reagents

The MCF7 cell line was maintained in RPMI supplemented with 5% fetal bovine serum (FBS), 5 µg/ml insulin, and antibiotic-antimycotic. The SUM159 cell line was maintained in Ham's F12 medium supplemented (with 5% FBS, 5 µg/ml insulin, 1 µg/ml hydrocortisone, and antibiotic/antimycotic 10,000 units/ml penicillin G sodium, 10,000 µg/ml streptomycin sulfate, and 25 µg/ml amphotericin B). LY294002, Akt inhibitor IV, and Bio were purchased from Millipore; perifosine was obtained from Keryx Biopharmaceuticals, and docetaxel (Taxotere) was from Sanofi Aventis.

Antibodies to phospho-Akt, Akt, β-catenin, GSK3-β, phospho-GSK3-β Ser^9^, PTEN, and phospho-PTEN Ser^380^ were purchased from Cell Signaling Technology. The α-tubulin antibody was from Santa Cruz Biotechnology, the ALDH1 antibody was from BD Transduction Laboratory, and the active-β-catenin (anti-ABC), clone 8E7 mouse monoclonal antibody was from Millipore Corporation.

The primary antibodies for human smooth muscle actin, Ki67, CK5/6, and CK18 were purchased from Zymed laboratories (Invitrogen).

### Dissociation of Normal Mammary Tissues

Mammary tissue from reduction mammoplasties was dissociated as previously described [19]. The single-cell suspension was used for various experiments including sphere formation, flow cytometry analyses, and lentiviral infections. At least three different patient samples (reduction mammoplasties) were used for each experiment involving NMECs.

### Mammosphere/Tumorsphere Assay

Single NMECs were plated on 1% agarose coated plates at a density of 1×10^5^ cells/ml and grown for 7–10 d. Subsequent cultures after dissociation of primary spheres were plated on ultra-low attachment plates at a density of 5×10^3^–1×10^4^ cells/ml. Mammosphere cultures were grown in a serum-free mammary epithelium basal medium as previously described [18]. Tumorspheres were cultured under identical conditions to mammospheres.

### Lentiviral Constructs and Infection of NMECs and Breast Cancer Cell Lines

All lentiviral constructs were prepared by the University of Michigan Vector or shRNA core facilities. The primers targeting the human PTEN short hairpin sequences were purchased from Integrated DNA Technologies. The forward primer TCCCAGGTGAAGGTATGTTCCTCCAATCTAAAGGATTGGAGGAATATATCTTCACCTGGGATTTTTTC and the reverse primer TCGAGAAAAAATCCCAGGTGAAGATATATTCCTCCAATCCTTTAGATTGGAGGAACATACCTTCACCTGGAC were digested with the Xho1 enzyme, annealed, and cloned into the pLentilox 3.7 vector. After confirmation of DNA sequences, the vectors were infected into 293 host cells to produce viruses at the University of Michigan Vector Core facility. Resulting lentiviral PTEN shRNA has been used to transfect NMECs and breast cancer cell lines. Two different CTNNB1 shRNA lentiviral vectors were purchased from University of Michigan shRNA core facility. TOP-GFP and FOP-GFP lentiviral reporter vectors were kindly provided by Irving L. Weissman (Stanford University School of Medicine, Stanford, California).

### Aldefluor Assay and Flow Cytometry

To measure and isolate cells with high ALDH activity, the Aldefluor assay was carried out according to manufacturer's (Stemcell Technologies) guidelines. Briefly, dissociated single cells from cell lines or from primary mammospheres were suspended in Aldefluor assay buffer containing an ALDH substrate, bodipy-aminoacetaldehyde (BAAA) at 1.5 µM, and incubated for 40 min at 37°C. To distinguish between ALDH-positive and -negative cells, a fraction of cells was incubated under identical condition in the presence of a 10-fold molar excess of the ALDH inhibitor, diethylamino benzaldehyde (DEAB). This results in a significant decrease in the fluorescent intensity of ALDH-positive cells and was used to compensate the flow cytometer.

### Lentivirus Infection of Cells

NMECs, SUM159, and MCF7 cell lines were infected with the lentivirus expressing human PTEN shRNA or control lentiviruses. The pLentilox 3.7 vector contains DsRed sequences in its backbone for selection. Following 12–16 h of incubation in suspension cultures of NMECs or monolayer cultures of breast cancer cell lines, the viruses were replaced with fresh medium.

### Implantation of Cells in NOD/SCID Mice

All mice were housed in the AAALAC-accredited specific pathogen-free rodent facilities at the University of Michigan. Mice were housed on sterilized, ventilated racks and supplied with commercial chow and sterile water both previously autoclaved. All experimentation involving live mice were conducted in accordance with standard operating procedures approved by the University Committee on the Use and Care of Animals at the University of Michigan. The fat pads of three week old NOD/SCID mice (NOD.CB17-Prkdcscid/J, stock number 001303) purchased from the Jackson Laboratories were cleared and replaced with 1:1 mixture of irradiated and nonirradiated human fibroblasts obtained from reduction mammoplasties. Within 2–3 wk, human fibroblasts generated a humanized mammary fat pad in mice. These resulting fat pads were injected with NMECs infected with DsRed or PTEN shRNA lentiviral vectors. A 60-d release 0.18-µg estrogen pellet (Innovative Research of America) was implanted subcutaneously in each mouse. The mice were killed after 4–8 wk and the fat pads were analyzed for outgrowths.

An identical protocol was followed for injection of breast cancer cell lines and xenografts, except that they were injected directly into the fat pads of NOD/SCID mice. Indicated dilutions of cell lines or xenografts were directly injected into fat pads of NOD/SCID mice. We used five mice for each experiment.

### Immunoblotting

Cells were lysed in Laemmli buffer, boiled, and subjected to SDS-PAGE. The proteins were transferred onto Nitrocellulose Membranes (Pierce) using semi dry Trans-Blot (Bio Rad Laboratories). Blots were first incubated in TBS blocking buffer containing either 2% milk or 2% BSA (for phospho-specific antibodies) for 1 or 2 h at room temperature and then with the respective primary antibodies diluted in TBST (containing 0.1% Tween20 and 2% BSA) either overnight at 4°C, or 2 h at room temperature. Subsequently, blots were washed and incubated with appropriate secondary antibodies (GE Healthcare) in TBST and detected using SuperSignal West Pico Chemiluminescent Substrate (Pierce).

### Immunostaining

For immunohistochemistry, paraffin-embedded sections were deparaffinized in xylene and rehydrated in graded alcohol. Antigen enhancement was done by incubating the sections in citrate buffer pH6 (Dakocytomation) as recommended. Staining was done using peroxidase histostain-Plus Kit (Zymed) according to the manufacturer's protocol. Sections were incubated with primary antibodies for 1 h. Following the incubation with broad-spectrum secondary antibody and the HRP-conjugated streptavidin, AEC (Zymed) was used as substrate for peroxidase. Slides were counter-stained with hematoxylin and coverslipped using glycerin.

For fluorescent staining, cells were fixed with 95% methanol at −20°C for 10 min. After rehydrating in PBS, cells were incubated with respective antibodies at room temperature for one hour, washed and incubated 30 min with FITC-conjugated secondary antibodies. The nuclei were stained with DAPI/antifade (Invitrogen) and coverslipped. Sections were examined with a fluorescent microscope (Leica).

### Statistical Analyses

Statistical differences for the number of mammospheres, GFP-positive cells, Aldefluor assays and tumor growths were determined using Students *t*-test.

## Supporting Information

Figure S1 **β-catenin and phospho-GSK3-β analyzed in Aldefluor-positive and Aldefluor-negative NMECs.** Aldefluor-positive cells express β-catenin with nuclear localization as compared to cytoplasmic β-catenin in Aldefluor-negative cells. Aldefluor-positive cells also showed higher expression of phospho-GSK3-β as compared to the Aldefluor-negative cells. Scale bars = 100 µm.
(0.41 MB TIF)

Figure S2 **PTEN knockdown increases normal mammary stem/progenitor cells.** (A) Mammary epithelial cells from reduction mammoplasties contain 5–7% Aldefluor-positive cells, and that increases to 15–18% in primary mammospheres. PTEN knockdown increases the Aldefluor-positive population in primary mammospheres as compared to that of control mammospheres. (B) Down-regulation of *PTEN* using lentiviral shRNA also maintains a higher level of Aldefluor-positive cells grown under adherent conditions. (C) Phospho**-**Akt expression measured by immunofluorescent staining in adherent versus mammosphere cultures from control and PTEN knockdown cells is shown. A higher Akt activity (p-Akt) was observed in cells with PTEN knockdown cultured in both adherent culture and mammospheres.
(0.53 MB TIF)

Figure S3 **Infection of NMECs with a lentiviral β-catenin shRNA produced a 50% reduction in the level of β-catenin protein expression as well as a significant reduction in active β-catenin as assessed by Western blotting.**
(0.06 MB TIF)

Figure S4 **MCF7 cells were analyzed for the side population as assessed by Hoechst dye exclusion, and approximately one million SP or non-SP cells were sorted.** Subsequently, SP or non-SP cells were analyzed by the Aldefluor assay. As indicated there was approximately a 2-fold enrichment of Aldelfuor-positive population in SP as compared to non-SP, which showed a similar percentage of Aldefluor-positive cells as seen in unfractionated MCF7 cells.
(0.19 MB TIF)

Figure S5 **PTEN knockdown activates Akt and accelarates tumor growth.** A) Representative tumors from MCF7-GFP control and MCF7-PTEN shRNA xenografts. (B) Tumor sections from MCF7-GFP and MCF7-PTEN shRNA stained with phospho-Akt antibodies demonstrated higher Akt phosphorylation in PTEN knockdown MCF7 xenografts. (C) Relative tumor growth of MCF7-GFP and MCF7-PTEN shRNA in NOD/SCID mice.
(0.94 MB TIF)

Figure S6 **Representative flow cytometry results of Aldefluor assays performed on primary SUM159, MC1 or UM2 tumor xenografts.** Treatment of cells with DEAB resulted in inhibition of ALDH1 activity. Following the compensation of the FITC channel based on DEAB inhibition, the cells were gated and the Aldefluor-positive cells were analyzed in the absence of the inhibitor. Perifosine or perifosine+docetaxel treated tumors displayed a 75–90% reduction in the Aldefluor-positive population as compared to the control or docetaxel treated SUM159, MC1, or UM2 tumors.
(0.59 MB TIF)

Figure S7 **Reimplantation of primary SUM159 and MC1 tumors treated with saline, docetaxel, perifosine, or both demonstrated different kinetics of tumor growth in secondary mice.** 50,000 or 10,000 cells from perifosine or the combination of perifosine- and docetaxel-treated mice produced a significant delay in growth in secondary mice. Moreover, 1,000 tumor cells from control or docetaxel-treated mice formed tumors when transplanted into secondary mice, while the same number of cells from perifosine or the combination of perifosine- and docetaxel-treated primary tumors failed to form secondary tumors.
(0.17 MB TIF)

Table S1 **Mammary outgrowths in humanized NOD/SCID mouse.** Serial dilutions of NMECs infected with DsRed or PTEN lentiviral shRNA were injected into the humanized mammary fat pads of NOD/SCID mice. Implantation of as few as 250 PTEN knockdown cells generated outgrowths in two out of four mice (*p*<0.01). In contrast, 5,000 DsRed-infected NMECs failed to generate outgrowths (*p*<0.005). In addition, PTEN knockdown cells formed larger outgrowths as compared to control DsRed cells.
(0.07 MB TIF)

## Abbreviations

ALDH: aldehyde dehydrogenase
Bio: 6-bromoindirubin-3′-oxime
ER: estrogen receptor
mTOR: mammalian target of rapamycin
NMEC: normal mammary epithelial cell
PTEN: phosphatase and tensin homolog
SP: side population
