# Supplementary figures and images for: Regulation of Mammary Stem/Progenitor Cells by PTEN/Akt/β-Catenin Signaling
Source: PLoS Biol. 2009 Jun 2;7(6):e1000121. doi: 10.1371/journal.pbio.1000121 (PMC2683567; doi:10.1371/journal.pbio.1000121)

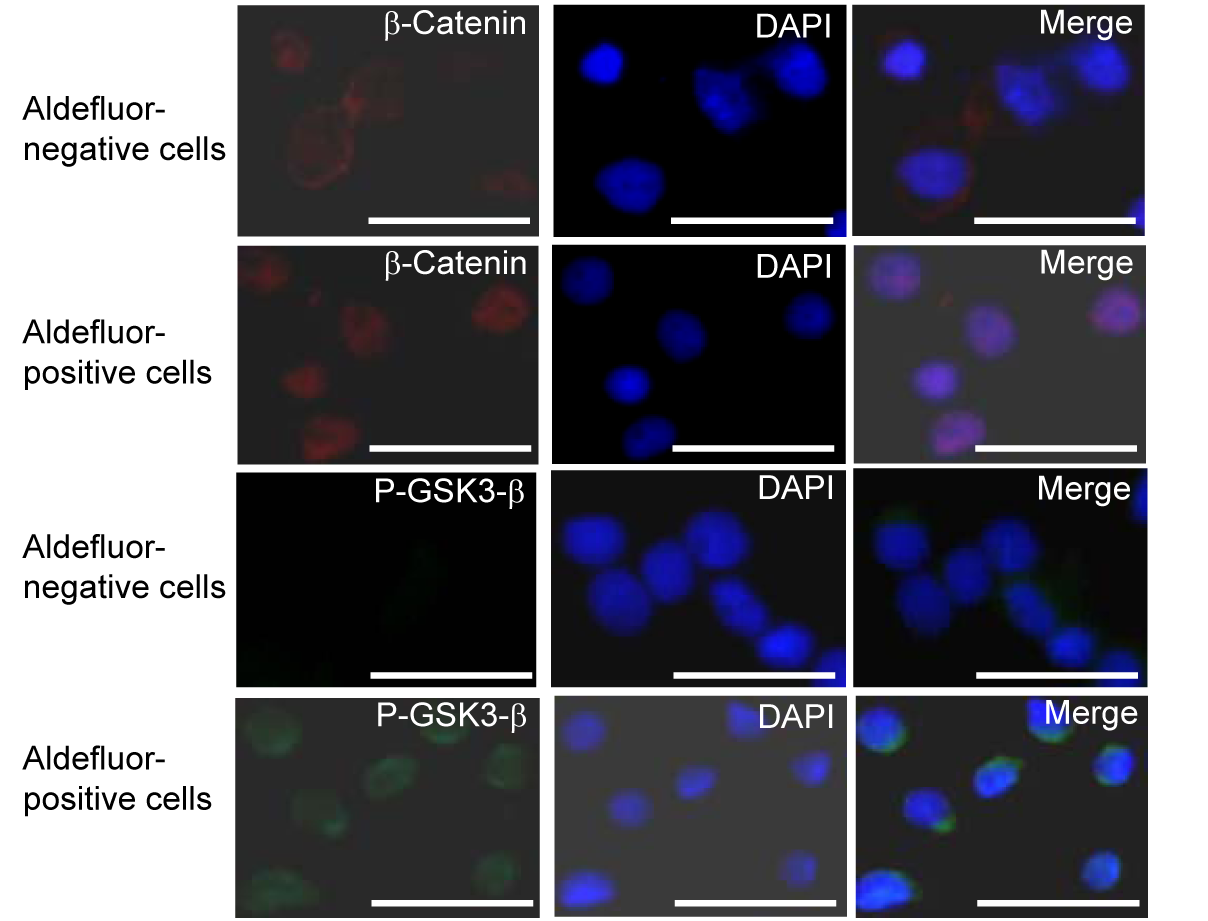

Supplement: Figure S1 — β-catenin and phospho-GSK3-β analyzed in Aldefluor-positive and Aldefluor-negative NMECs. Aldefluor-positive cells express β-catenin with nuclear localization as compared to cytoplasmic β-catenin in Aldefluor-negative cells. Aldefluor-positive cells also showed higher expression of phospho-GSK3-β as compared to the Aldefluor-negative cells. Scale bars = 100 µm. (0.41 MB TIF) [file pbio.1000121.s001.tif]

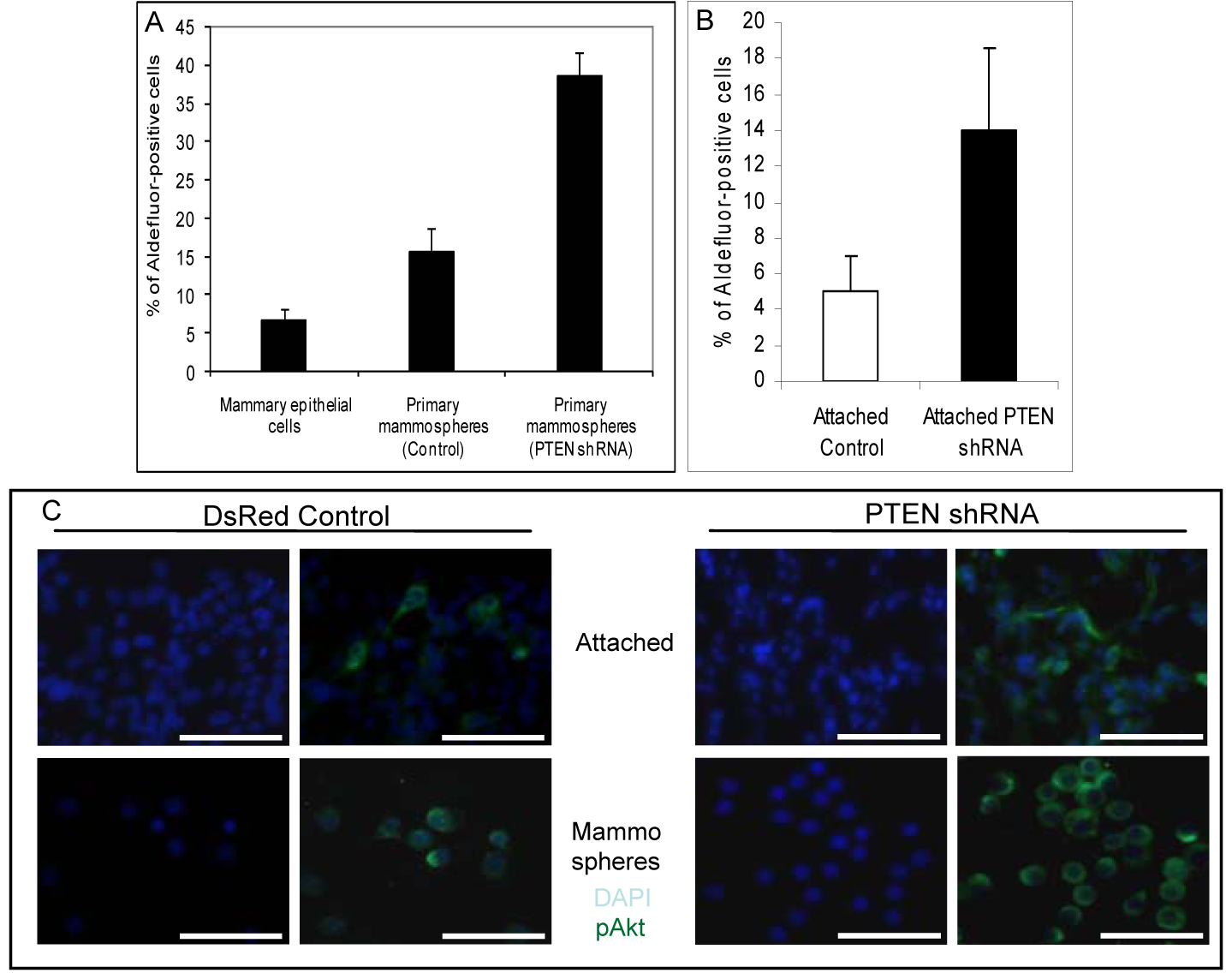

Supplement: Figure S2 — PTEN knockdown increases normal mammary stem/progenitor cells. (A) Mammary epithelial cells from reduction mammoplasties contain 5–7% Aldefluor-positive cells, and that increases to 15–18% in primary mammospheres. PTEN knockdown increases the Aldefluor-positive population in primary mammospheres as compared to that of control mammospheres. (B) Down-regulation of PTEN using lentiviral shRNA also maintains a higher level of Aldefluor-positive cells grown under adherent conditions. (C) Phospho-Akt expression measured by immunofluorescent staining in adherent versus mammosphere cultures from control and PTEN knockdown cells is shown. A higher Akt activity (p-Akt) was observed in cells with PTEN knockdown cultured in both adherent culture and mammospheres. (0.53 MB TIF) [file pbio.1000121.s002.tif]

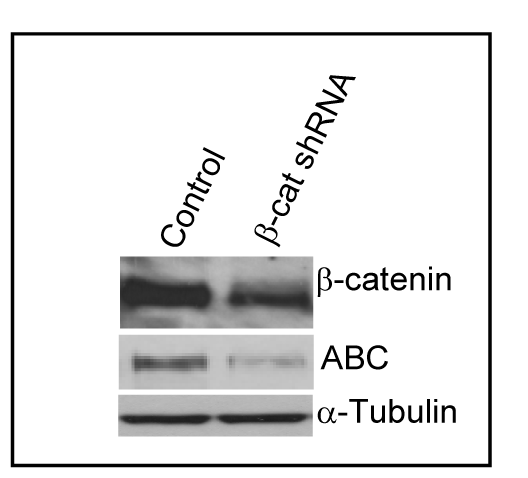

Supplement: Figure S3 — Infection of NMECs with a lentiviral β-catenin shRNA produced a 50% reduction in the level of β-catenin protein expression as well as a significant reduction in active β-catenin as assessed by Western blotting. (0.06 MB TIF) [file pbio.1000121.s003.tif]

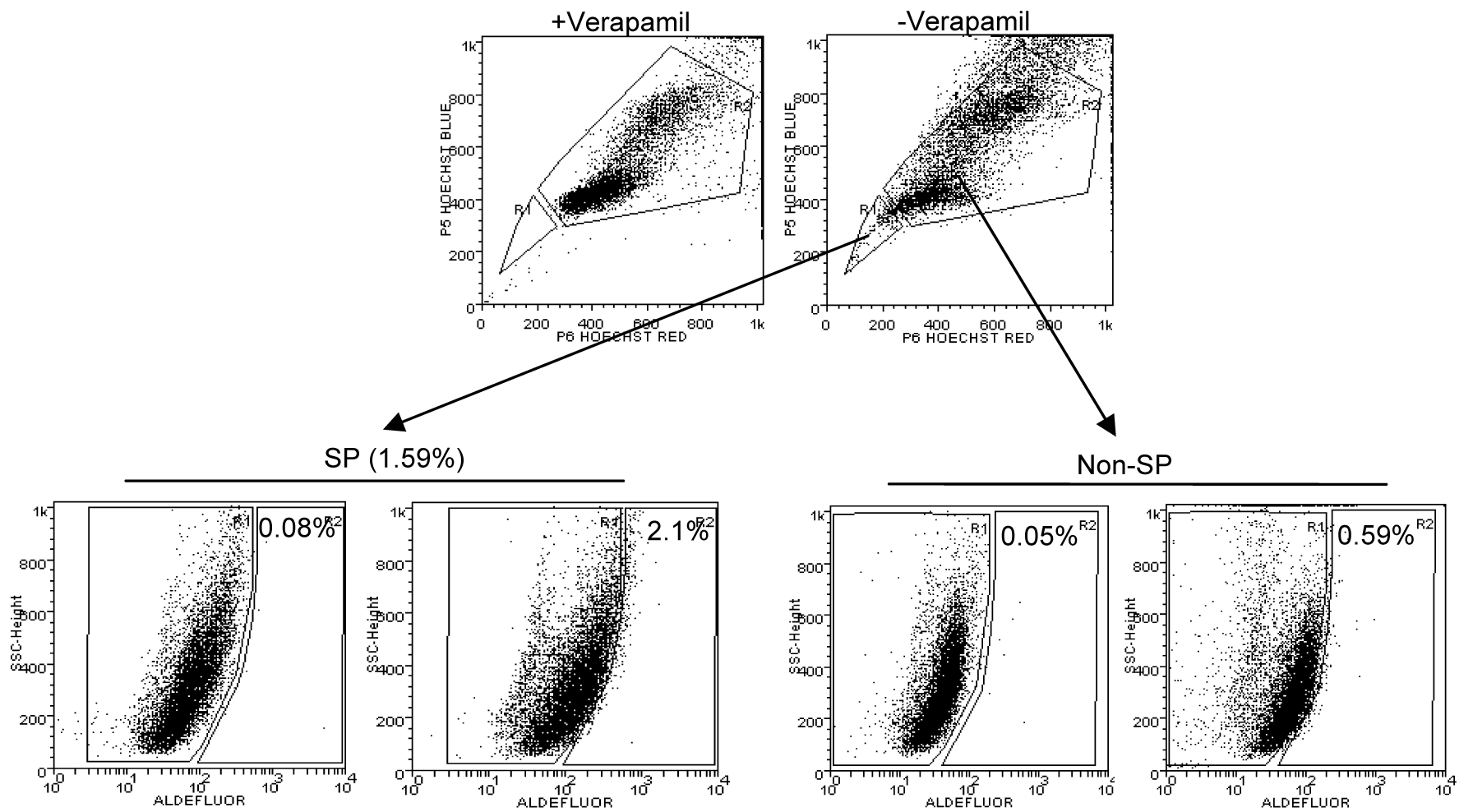

Supplement: Figure S4 — MCF7 cells were analyzed for the side population as assessed by Hoechst dye exclusion, and approximately one million SP or non-SP cells were sorted. Subsequently, SP or non-SP cells were analyzed by the Aldefluor assay. As indicated there was approximately a 2-fold enrichment of Aldelfuor-positive population in SP as compared to non-SP, which showed a similar percentage of Aldefluor-positive cells as seen in unfractionated MCF7 cells. (0.19 MB TIF) [file pbio.1000121.s004.tif]

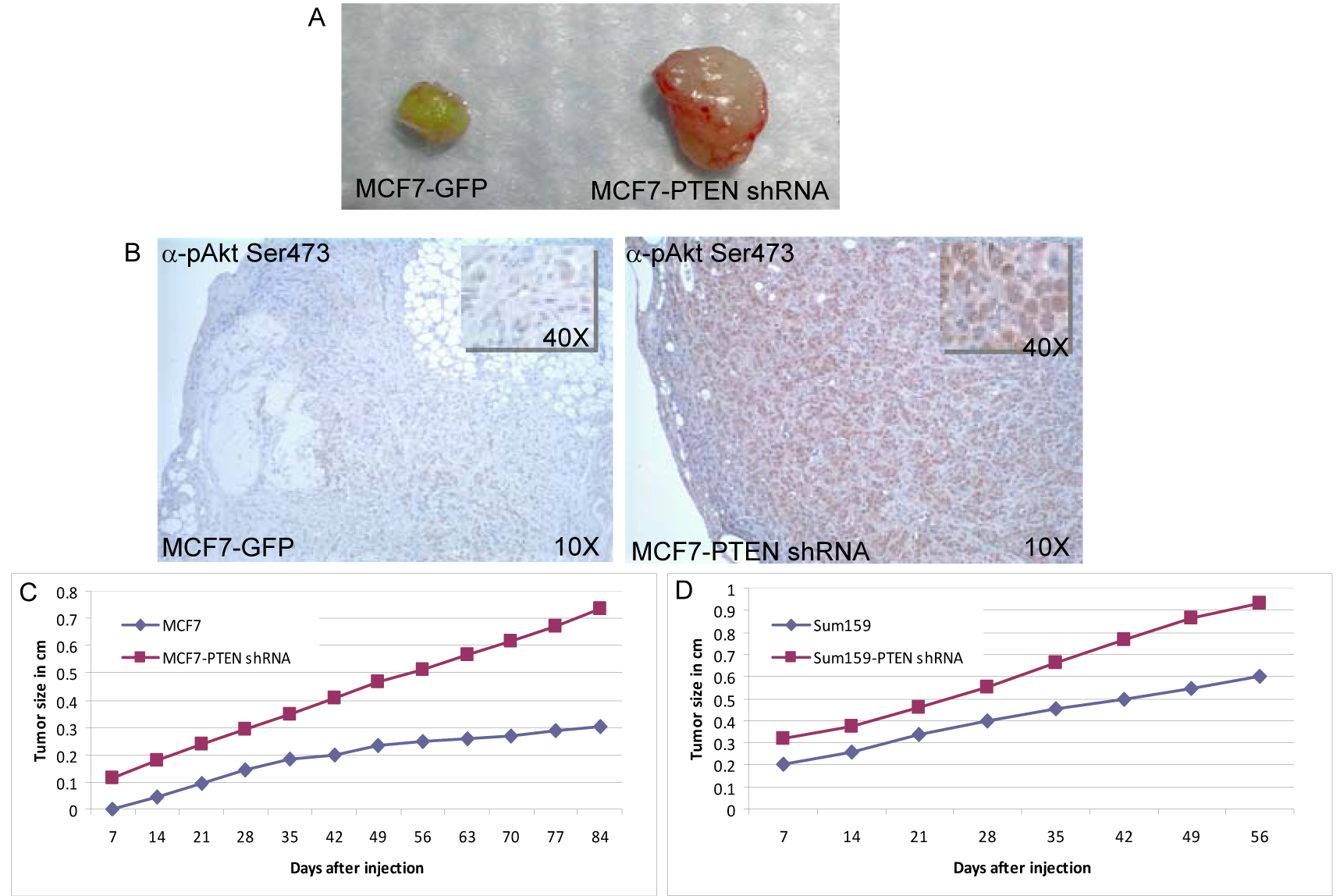

Supplement: Figure S5 — PTEN knockdown activates Akt and accelarates tumor growth. A) Representative tumors from MCF7-GFP control and MCF7-PTEN shRNA xenografts. (B) Tumor sections from MCF7-GFP and MCF7-PTEN shRNA stained with phospho-Akt antibodies demonstrated higher Akt phosphorylation in PTEN knockdown MCF7 xenografts. (C) Relative tumor growth of MCF7-GFP and MCF7-PTEN shRNA in NOD/SCID mice. (0.94 MB TIF) [file pbio.1000121.s005.tif]

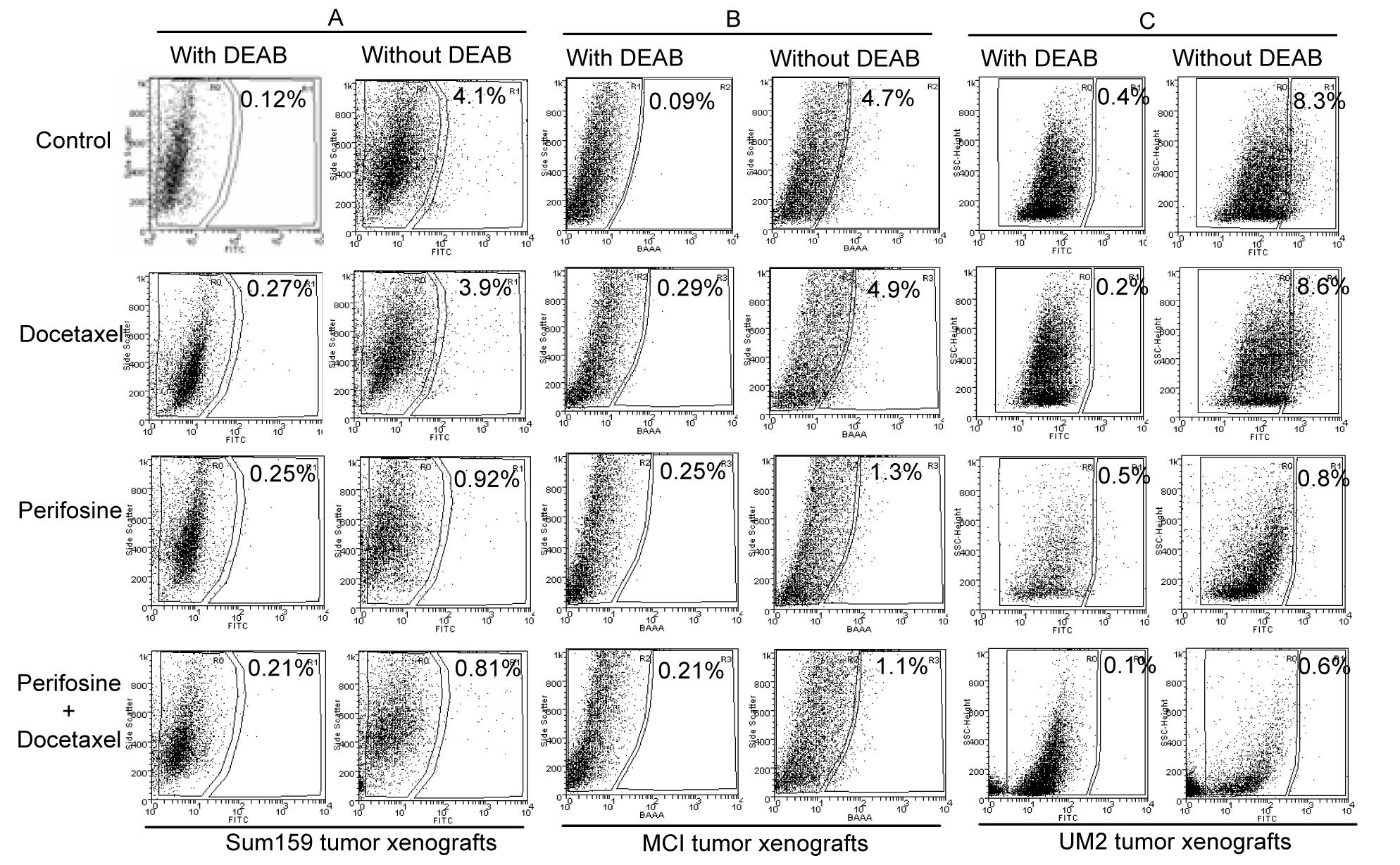

Supplement: Figure S6 — Representative flow cytometry results of Aldefluor assays performed on primary SUM159, MC1 or UM2 tumor xenografts. Treatment of cells with DEAB resulted in inhibition of ALDH1 activity. Following the compensation of the FITC channel based on DEAB inhibition, the cells were gated and the Aldefluor-positive cells were analyzed in the absence of the inhibitor. Perifosine or perifosine+docetaxel treated tumors displayed a 75–90% reduction in the Aldefluor-positive population as compared to the control or docetaxel treated SUM159, MC1, or UM2 tumors. (0.59 MB TIF) [file pbio.1000121.s006.tif]

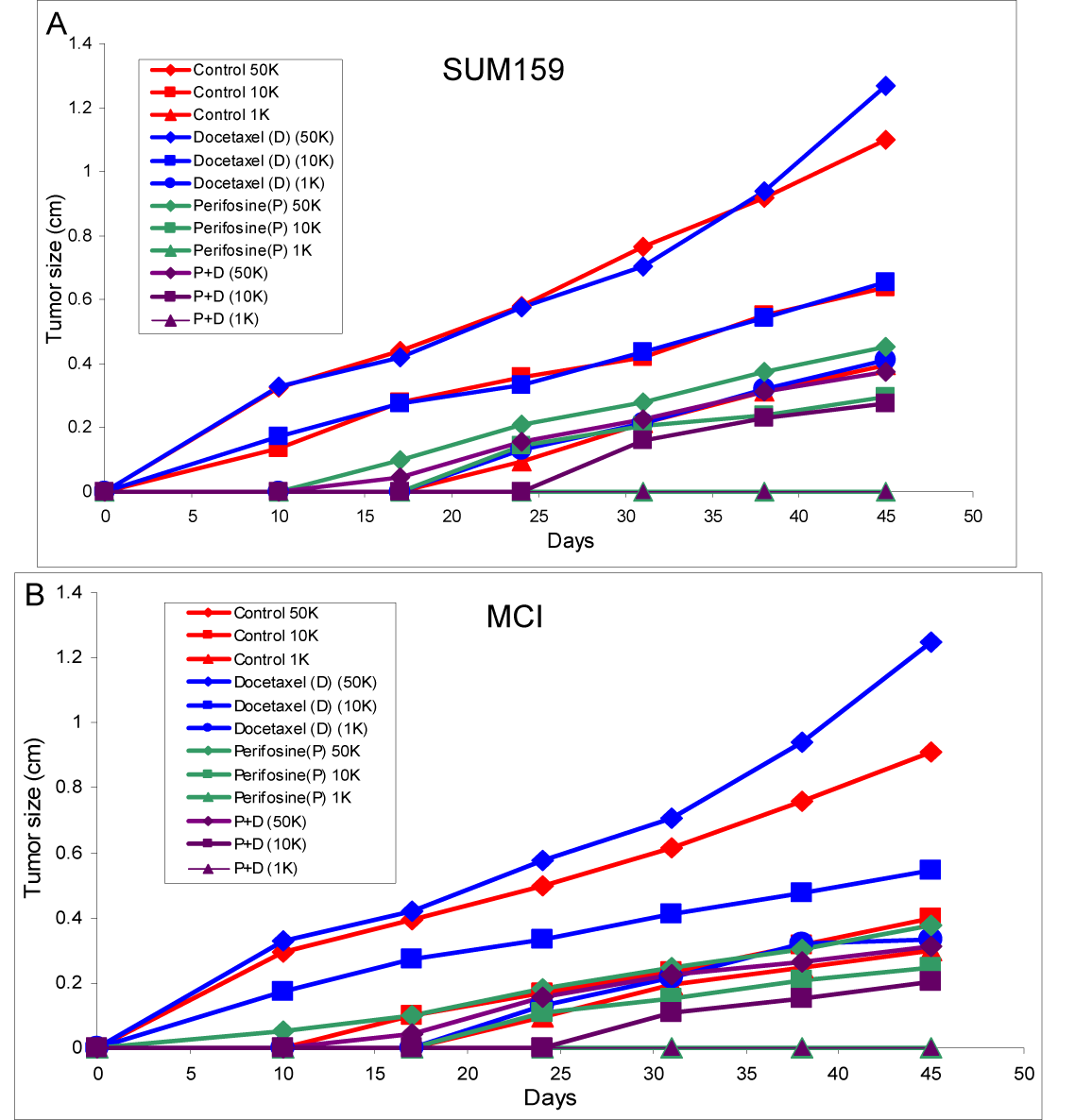

Supplement: Figure S7 — Reimplantation of primary SUM159 and MC1 tumors treated with saline, docetaxel, perifosine, or both demonstrated different kinetics of tumor growth in secondary mice. 50,000 or 10,000 cells from perifosine or the combination of perifosine- and docetaxel-treated mice produced a significant delay in growth in secondary mice. Moreover, 1,000 tumor cells from control or docetaxel-treated mice formed tumors when transplanted into secondary mice, while the same number of cells from perifosine or the combination of perifosine- and docetaxel-treated primary tumors failed to form secondary tumors. (0.17 MB TIF) [file pbio.1000121.s007.tif]

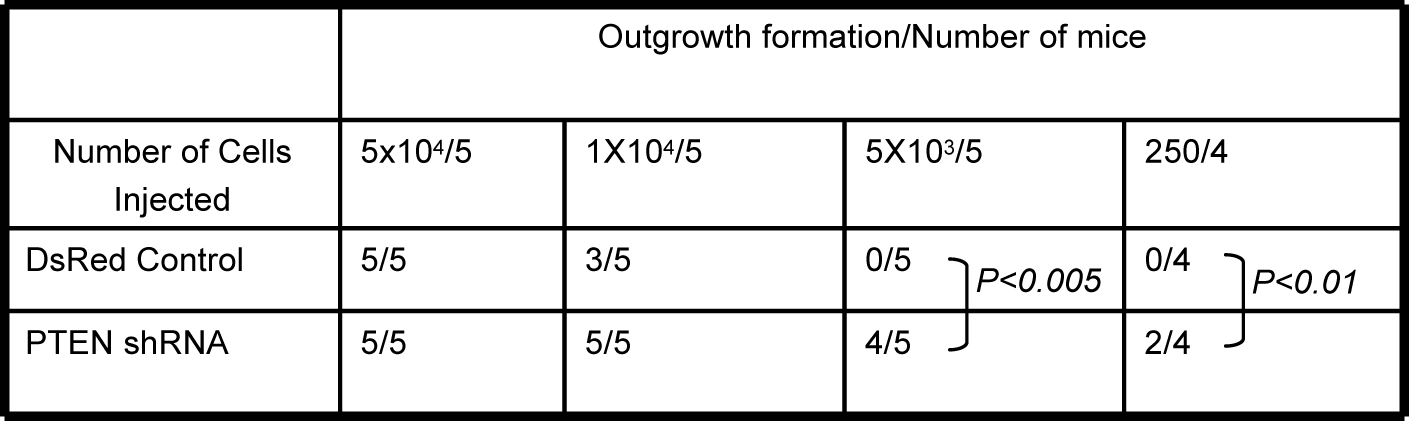

Supplement: Table S1 — Mammary outgrowths in humanized NOD/SCID mouse. Serial dilutions of NMECs infected with DsRed or PTEN lentiviral shRNA were injected into the humanized mammary fat pads of NOD/SCID mice. Implantation of as few as 250 PTEN knockdown cells generated outgrowths in two out of four mice (p<0.01). In contrast, 5,000 DsRed-infected NMECs failed to generate outgrowths (p<0.005). In addition, PTEN knockdown cells formed larger outgrowths as compared to control DsRed cells. (0.07 MB TIF) [file pbio.1000121.s008.tif]
